# Population pharmacokinetics, dosing optimization and clinical outcomes of biapenem in patients with sepsis

**DOI:** 10.3389/fphar.2024.1388150

**Published:** 2024-05-10

**Authors:** Dayu Chen, Xuanyu Wu, Haixia Zhang, Huimin Yao, Lu Jin, Xuemei Luo, Jinchun Liu, Zejun Wu, Yuanchen Li, Wei Xu, Weihong Ge, Xingkai Chen, Huaijun Zhu

**Affiliations:** ^1^ Department of Pharmacy, Nanjing Drum Tower Hospital, Nanjing, China; ^2^ Nanjing Medical Center for Clinical Pharmacy, Nanjing Drum Tower Hospital, Nanjing, China; ^3^ Department of Pharmacy, Nanjing Drum Tower Hospital, Nanjing Drum Tower Hospital Clinical College of Nanjing University of Chinese Medicine, Nanjing University of Chinese Medicine, Nanjing, China; ^4^ School of Pharmacy, Faculty of Medicine, Macau University of Science and Technology, Taipa, Macau SAR, China; ^5^ Department of Cardiology, Nanjing Drum Tower Hospital, Nanjing Drum Tower Hospital Clinical College of Nanjing University of Chinese Medicine, Nanjing University of Chinese Medicine, Nanjing, China; ^6^ Department of Pharmacy, Nanjing Drum Tower Hospital, School of Basic Medicine and Clinical Pharmacy, China Pharmaceutical University, Nanjing, China

**Keywords:** biapenem, pharmacokinetics, dosing regimen, sepsis, Monte Carlo simulation

## Abstract

**Introduction:** Biapenem is a carbapenem antibiotic widely used in Asia, can be used for the treatment of adults and children with infections due to susceptible bacteria. Although biapenem is utilized in the treatment of a diverse range of bacterial infections, current pharmacokinetic data in the context of septic populations remain limited. Consequently, our research aims to evaluate the pharmacokinetics and efficacy of biapenem within a septic population to optimize biapenem therapy.

**Methods:** In this study, we characterized the pharmacokinetics of biapenem in septic patients using a population pharmacokinetic (PPK) approach. The clinical PK data to develop the PPK model were obtained from 317 septic patients admitted to Nanjing Drum Tower Hospital between 2018 and 2022. All patients were randomized to the modeling and validation cohorts at a 3:1 ratio, with PPK modeling and validation performed utilizing the NONMEM software.

**Results:** The model found to best describe the available data was a two-compartment PPK model with first-order elimination characterized by the parameters clearance (CL), central volume (V1), peripheral volume (V2), and intercompartmental clearance (Q). A covariate analysis identified that creatinine clearance (CLCR) was a significant covariate influencing biapenem CL, while blood urea nitrogen (BUN) was a significant covariate influencing biapenem Q. Accoding to the clinical outcome analyses, 70% of the time that the free antimicrobial drug concentration exceeds the MIC (*fT*_>MIC_) is associated with favourable clinical outcomes. The PPK model was then used to perform Monte Carlo simulations to evaluate the probability of attaining 70% *fT*_>MIC_.

**Conclusions:** A final PPK model of biapenem was established for patients with sepsis. The current daily dosage regimen of 1.2 g may insufficient to achieve 70% *fT*_>MIC_ in septic patients. The dosage regimen of 600 mg every 6 h appears to be the optimal choice.

## 1 Introduction

Biapenem (BPM) is a carbapenem antibiotic, employed in the combat against bacterial infections (Brismar et al., 1996). It exhibits its antibacterial prowess by inhibiting bacterial cell wall synthesis. Comparable to the *in vitro* potency of imipenem and meropenem, BPM possesses a broad-spectrum efficacy, spanning Gram-negative and Gram-positive bacteria, and extending to anaerobic bacteria (Dong et al., 2016). Demonstrations of both clinical and bacteriological effectiveness have been evident in the management of diverse bacterial infections - pneumonia, complicated urinary tract infections, pyelonephritis, peritonitis, and others (Perry and Ibbotson, 2002). Over the past decades, BPM has ascended to become a choice in treating infections precipitated by extended-spectrum β-lactamase-producing Gram-negative bacteria (Alonso et al., 2006).

Alterations in the volume of distribution (Vd) and clearance of antibiotics have been observed in sepsis (van der Poll, 2001; Roberts and Lipman, 2006). Endotoxins induce the synthesis of various endogenous mediators, including cytokines, interleukins, and platelet-activating factor, can influence the vascular endothelium, leading to vasodilation, along with an aberrant distribution of blood flow, endothelial damage, and heightened capillary permeability. This syndrome results in fluid translocation from the intravascular compartment to the interstitial space, consequently increasing the Vd of water-soluble drugs, including carbapenems, and diminishing their serum concentrations (Murínová et al., 2022). It was reported that bipenem has a relatively short elimination half-life of approximately 1 hour. Additionally, it manifests a significant inter-individual variability in its pharmacokinetic characteristics, which leads to substantial disparities in the blood drug concentration amongst patients. Consequently, identical dosing regimens can produce disparate clinical outcomes in different patients. Sole dependence on the suggested dosing regimen outlined in the product label is inadequate for dose adjustments in diverse patient populations, particularly in cases involving sepsis (Evans et al., 2021). Therefore, exploration into the pharmacokinetic profiles of BPM within the septic patient populace is crucial in order to predict the pharmacokinetic parameters of this specific populace, and to effectively enhance or refine the dosage for improved patient outcomes.

BPM exhibits a time-dependent bactericidal action, where its antimicrobial effectiveness is contingent upon the duration for which serum drug levels surpass the minimal inhibitory concentration (MIC). In the current clinical practice, BPM is commonly administered twice or three times a day, with a dose of 300mg–600 mg per administration (Griffith et al., 2023). However, due to its relatively brief elimination half-life and time-dependent nature, achieving the pharmacokinetics/pharmacodynamics (PK/PD) target remains challenging with dosing intervals of either 8 or 12 h (Ikawa et al., 2008a; Ikawa et al., 2008b; Rao et al., 2023).

Currently, there is a notable gap in the literature, as population pharmacokinetic studies focusing on BPM in septic patients are lacking. This is particularly significant considering the frequent prescription of BPM within this patient population. Thus, the primary objective of this study is to investigate the pharmacokinetics of BPM in septic individuals utilizing a population pharmacokinetic (PPK) approach. This investigation is intended to facilitate dosing optimization, thereby enhancing the achievement of the PK/PD target in this patient cohort.

## 2 Materials and methods

### 2.1 Study design, patient enrollment and data collection

This was a retrospective, single center study performed in Nanjing Drum Tower hospital, a university-affiliated tertiary medical center with 3,800 beds. The study was performed in accordance with the ethical standards of the Declaration of Helsinki. The study was approved by the Ethics Committee of Nanjing Drum Tower Hospital Affiliated Hospital of Nanjing University Medical School, Nanjing, China (2022-504-01) and was registered at the Chinese Clinical Trial Registry (ChiCTR2300073976). The need for informed consent was waived by the ethics committee in view of the observational and retrospective nature of the study. Adult patients (over 18 years of age) with sepsis who treated by BPM in Nanjing Drum Tower Hospital (China) from January 2018 to May 2022 were included. Sepsis was defined by criteria according to the Third International Consensus Definitions for Sepsis and Septic Shock (Singer et al., 2016). The exclusion criteria were as follows: patients who received BPM for less than 48 h; patients received renal replacement therapy or extracorporeal membrane oxygenation during BPM therapy; patients did not receive therapeutic drug monitoring (TDM) and patients with incomplete medical record. Given the nature of this retrospective observational study, no intervention was made to standardize care. According to the clinical practice, samples were usually collected after the third dose interval of BPM and were measured for serum concentration.

Clinical records of all included patients were reviewed and evaluated. BPM dosing regimens, including administration times and infusion rates were collected. The initiation and termination times of each drug infusion, along with the timing for blood sampling, were meticulously documented within the hospital’s electronic medical records system via an automated information system, with time entries recorded with minute-level precision. The gender, age, body weight, alanine aminotransferase (ALT), aspartate transaminase (AST), alkaline phosphatase (AKP), glutamyltransferase (GGT), total bilirubin (TB), direct bilirubin (DB), total protein (TP), albumin (ALB), blood urea nitrogen (BUN), serum creatinine (SCR), white blood cell count (WBC), hemoglobin (HB), platelet (PLT) of included patients during BPM therapy were recorded. The estimated glomerular filtration rate (eGFR) was calculated according to the Modification of Diet in Renal Disease formula and creatinine clearance (CLCr) was calculated according to the Cockcroft-Gault formula (Cockcroft and Gault, 1976). Body mass index (BMI) was calculated as body weight (kg) divided by the square of height (m). Immunocompromised patients, severity of sepsis and primary infection site were also included for analysis. All patients were randomly divided into a modeling cohort and an validation cohort in a ratio of 3:1.

### 2.2 Dosing, sampling schedule, bioanalytical method and determination of BPM concentration

TDM of BPM is performed routinely in septic patients according to the clinical practice at the participating site. According to the product label, the dose of BPM for adults was 300–600 mg per administration, given 2–4 times per day, with a daily dose not exceeding 1.2 g. BPM was intravenously administered to all patients with 100 mL of sodium chloride or glucose injection as a solvent. BPM were infused over 1 h according to the clinical practice.

Blood samples were obtained via an indwelling cannula, collected into EDTA tubes, and immediately placed on ice during transferring to the drug monitoring laboratory. In order to stabilize BPM, all blood samples were then pretreatmented by adding an equal volume of 1 M 3- (*N*-morpholino) propanesulfonic acid (MOPS) buffer (pH 7.0) as a stabilizer after centrifugation. The samples were then frozen and stored prior to assay at −20°C. After samples were mixed with their internal standard (50 mg/mL 5-hydroxyindole-3-acetic acid) and transferring to an ultrafiltration device and centrifuged, the plasma concentration of BPM was then measured by a Shimadzu LC-2030C 3D High-Performance Liquid Chromatography Analyzer (Shimadzu Corporation, Tokyo, Japan) with a reversed-phase column (TSKgel ODS-100V, 4.6 mm × 250 mm, 5µm; Tosoh Corporation, Yamaguchi-ken, Japan). The ultraviolet absorbance was detected at 300 nm. The mobile phase was a mixture of potassium dihydrogen phosphate (pH 6.5–7.0) and methanol (94:6). To investigate the linear relationship, 270 μL of blank plasma was taken and subsequently fortified with BPM standard solutions at concentrations of 3, 6, 18, 60, 180, and 300 mg/L, each at a volume of 30 μL. This resulted in final BPM plasma concentrations of 0.3, 0.6, 1.8, 6.0, 18.0, and 30.0 mg/L. The samples were processed according to the method described before, and the chromatographic peaks were recorded. The concentration of BPM (x) was plotted on the *x*-axis, while the ratio of the peak area of BPM (A_s) to the internal standard peak area (A_i) was plotted on the *y*-axis. Weighted least squares regression [with the weight coefficient being (1/C^2 was employed for linear regression to establish the standard curve. The resulting regression equation was (y = 0.4094x - 0.0665) (with r = 0.9999), indicating a strong linear relationship between the plasma concentration of BPM and the peak area within the range of 0.3–30 μg/mL, with 0.3 μg/mL as the lower limit of quantitation. To assess the precision and accuracy of the method, blank plasma samples were prepared with BPM concentrations of 0.3, 0.6, 3, and 24 mg/L. Each concentration level had a set of 5 samples. These samples were processed and analyzed as per the protocol outlined before. The chromatographic peak areas were recorded, and the concentrations of each sample were calculated using the established standard curve. This process was repeated across three batches to evaluate the method’s precision and accuracy. For the stability assessment, 270 μL of blank plasma was fortified with BPM standard solutions to achieve concentrations of 0.3, 0.6, 3, and 24 mg/L. Additionally, 100 μL of stabilizer and 20 μL of internal standard solution were added to each sample. The stability of BPM in plasma was examined under various conditions: room temperature storage for 2 h, room temperature storage for 6 h, three freeze-thaw cycles, and storage at −80°C for 60 days. Concentrations of BPM in plasma were calculated using the standard curve and compared with theoretical concentrations. The relative deviations of all measurements were within 15%, indicating that the plasma samples remained stable under the tested conditions of room temperature storage for both 2 and 6 h, three freeze-thaw cycles, and long-term storage at −80°C for 60 days. The intra- and inter-day coefficients of variation (CVs) were all <15%.

### 2.3 Population pharmacokinetic model development

The non-linear mixed effect modeling software NONMEM Ver. 7.3 (Icon Inc., Mayfield, PA, United States) compiled with gFortran (Version 4.6; http://www.gfortran.org) was used to perform the population pharmacokinetic analysis of BPM with the first-order method throughout the model-building procedure. GraphPad Prism 9.2 (GraphPad Software, La Jolla, CA, United States), R (Version 3.6.1; http://www.r-project.org) and the Xpose package (Version 4.5.3; http://xpose.sourceforge.net)were used for visual diagnosis.

#### 2.3.1 Basic model

The structural pharmacokinetics model composed of the one or two compartment with first-order elimination were compared. The variability among subjects was quantified by assuming that individual parameters followed a multivariate log-normal distribution, and the inter-individual variability of patients was described using an exponential model (Eq. 1):

$$Pi=TV\left(P\right)*\exp\left(\eta i\right)$$ (1)

where P i is the individual parameter estimate for the i -th patient, TV(P) represents the typical population value of pharmacokinetic parameters. ηi is the inter-individual variability with a mean of 0 and variance of ω^2^.

The intra-individual variability of pharmacokinetic parameters in patients is described using an additive model (Eq. 2), exponential model (Eq. 3), or a combined additive and exponential model (Eq. 4):

Y=IPRED+ε (2)

$$Y=IPRED\times \left(1+\varepsilon \right)$$ (3)

$$Y=IPRED\times \left(1+\varepsilon 1\right)+\varepsilon 2$$ (4)

where Y represents the observed blood drug concentration, IPRED is the individual predicted concentration, and ε is the residual variable with a mean of 0 and variance of σ^2^.

#### 2.3.2 Covariate models and model selection criteria

All covariates were introduced into the basic model in both linear and nonlinear methods, and the significance of each covariate was evaluated through likelihood ratio tests and visual inspection of diagnostic goodness-of-fit plots. The continuous covariates, including age, weight, ALT, AST, AKP, GGT, TB, DB, TP, ALB, BUN, SCR, eGFR, CLCr, WBC, HB, PLT, WBC,HB, PLT and categorical covariate gender, immunocompromisation, septic shock and primary infection site were screened for their influence on clearance and the volume of distribution. A model was established using a forward inclusion approach, with each covariate being individually added to the basic model for analysis. After that, a backward elimination approach was used. If the introduction of a covariate to the model decreased the objective function value (OFV) more than 3.84 (*df* = 1, *p* < 0.05), the covariate was considered to have a significant impact on the model and was included. Conversely, if the OFV did not significantly decrease, the covariate was excluded. This process was repeated until the covariate model was initially established with no significant reduction in the OFV. The backward elimination step was applied to examine the covariates included in the model. If the removal of a covariate resulted in an increase in the OFV less than 6.63 (*df* = 1, *p* < 0.01), the covariate was considered to have no statistical significance and was removed from the full regression model. The final model was established after this process was repeated for each covariate until the change in the OFV value was greater than 6.63.

#### 2.3.3 Model evaluation

Internal evaluation of the model was then performed. The goodness-of-fit (GOF) plots of the model including: the observed concentrations (DV) versus population prediction (PRED), DV versus individual prediction (IPRED), conditional weighted residuals (CWRES) versus PRED and CWRES versus time were plotted (Hooker et al., 2007). A prediction corrected visual predictive check (pcVPC) was used to describe the predictive performance of the model based on the distribution characteristics of observed and predicted concentrations in the graph, as well as the proportion of observed concentrations falling within the 95% confidence interval (CI) of the predicted concentrations. To evaluate the stability and reliability of the final model, 1,000 independent repeated samples were performed by using the non-parametric bootstrap method, comparing the 95% CI of the sampling results with the parameter estimates of the model.

One-quarter of the patients were included in the validation cohort. The PRED were estimated and compared with the corresponding observations by estimating the relative prediction error (PE) using Eq. 5. The median prediction error (MDPE) (Eq. 6), median absolute prediction error (MAPE) (Eq. 7) were used to evaluate the predictive accuracy and precision. PE within ±20% (F_20_) (Eq. 8) and PE within ±30% (F_30_) (Eq. 9) were calculated to evaluate the accuracy and precision of the model. In this study, when the standards of MDPE ≤±15%, MAPE ≤30%, F_20_ > 35%, and F_30_ > 50% were attained after evaluated by validation cohort, the model was considered to be satisfactory, clinically acceptable and have decent predictive performance (Bleeker et al., 2003; Nguyen et al., 2017).

$$PEi=\frac{PREDi-DVi}{DVi}\times 100\%$$ (5)

$$MDPE=Median\left(\frac{PREDi-DVi}{DVi}\right)\times 100\%$$ (6)

$$MAPE=Median\left(\left|\frac{PREDi-DVi}{DVi}\right|\right)\times 100\%$$ (7)

$$F_{20}=\frac{N\left(\left|PE\right|\leq 20\%\right)}{N\left(DV\right)}\times 100\%$$ (8)

$$F_{30}=\frac{N\left(\left|PE\right|\leq 30\%\right)}{N\left(DV\right)}\times 100\%$$ (9)

### 2.4 Microbial efficacy and clinical outcomes

All antimicrobial susceptibility tests were conducted at the Microbiology Laboratory at Nanjing Drum Tower Hospital. The MIC values of all isolates were determined following the recommendations of The Clinical and Laboratory Standards Institute by means of agar dilution method in our bacteriological laboratory for each patient in whom the microorganism was identified.

Clinical outcomes were compared in patients with at least one pathogen was identified. The primary treatment outcome was the clinical response, which was classified as either success or failure. Clinical success was defined as the resolution or improvement of infection-related clinical signs and symptoms as well as the microbial eradication at the end of BPM therapy with no need to add or change the antibacterial therapy. Clinical failure was defined as the persistence or worsening of any clinical signs or symptoms of infection, the emergence of any new clinical signs or symptoms of infection, or the need for additional systemic antibacterial medication, or the failure to achieve microbial eradication at the end of BPM therapy.

### 2.5 Dosing regimen optimization based on a pharmacokinetic model

Carbapenems are classified as time-dependent antimicrobial agents, characterized by the pharmacodynamic parameter of time exceeds the MIC (*fT*_>MIC_). The PK/PD index associated with optimal carbapenem activity is the % *fT*_>MIC_ (40%–70%) according to current guidelines (Li et al., 2007; McKinnon et al., 2008; Abdul-Aziz et al., 2020). Monte Carlo simulations (n = 10,000) were performed using the NONMEM in order to optimize the dose strategy of BPM. The final PPK model was used to perform Monte Carlo simulations to evaluate the probability of target attainment (PTA). From the simulated concentration-time profiles, *fT*_>MIC_ was determined for each virtual patient over a range of MIC values, from 0.0625 to 2.0 mg/L. Then, the PTA was calculated as the percentage of patients who achieved 70% *fT*_>MIC_ targets, in order to optimize BPM therapy.

Subsequent deescalation to other narrow-spectrum antibiotics, according to microbiological results, was considered a standard of care and did not imply treatment failure. The secondary treatment outcome was ICU mortality.

## 3 Results

### 3.1 Patients

According to the predetermined patient inclusion criteria of this study, a total of 466 BPM measurements collected from 317 adult patients were included. Among them, 351 blood concentration samples collected from 245 patients were defined as the modeling cohort, while the other 115 BPM concentration samples collected from 72 patients were defined as the external evaluation cohort. Each participant received an average of 1.5 samples. Supplementary Figure S1 in the Supplementary Material presents the scatter plots of time versus BPM concentration. The median trough drug concentration (C_min_) from the modeling and external evaluation datasets was 2.1 mg/L and 1.8 mg/L, respectively. Clinical characteristics of the patients included in the analysis are summarized in Table 1.

**TABLE 1:** Clinical characteristics.

Variable	Modeling cohort (n = 245)			External evaluation cohort (n = 72)		
	Mean ± SD	Median (range)	N (%)	Mean ± SD	Median (range)	N (%)
Gender						
Male			156 (63.7)			46 (63.9)
Female			89 (36.3)			26 (36.1)
Age (years)	59.13 ± 19.01	63 (18–97)		58.18 ± 14.53	60 (18–85)	
Weight (kg)	63.40 ± 11.34	62 (36.8–100)		63.78 ± 15.90	60.5 (45–148)	
ALT (U/L)	51.23 ± 161.04	20.5 (0.8–2,813.3)		46.19 ± 89.92	19.8 (4.7–842.3)	
AST (U/L)	69.05 ± 342.55	22.3 (4.9–6,598)		58.70 ± 176.25	24.4 (4.6–2002.2)	
AKP (U/L)	104.62 ± 83.65	79.1 (14.9–816)		141.14 ± 174.57	87.2 (35.5–1,576.1)	
GGT (U/L)	76.21 ± 89.59	45.7 (5.1–758.8)		123.24 ± 228.58	51.05 (9.1–1,536.7)	
TB (μmol/L)	20.88 ± 40.76	11.2 (1.6–453.7)		37.64 ± 63.84	16.2 (2.7–393.1)	
DB (μmol/L)	9.84 ± 26.13	3.3 (0.1–320.7)		22.35 ± 45.05	5.5 (0.3–277)	
TP (g/L)	59.90 ± 9.82	59.8 (21.1–116.4)		59.66 ± 10.31	59.3 (39.7–123.7)	
ALB (g/L)	34.68 ± 5.30	34.85 (3.5–55.1)		34.67 ± 4.52	34.9 (19.1–44.7)	
BUN (mmol/L)	9.84 ± 8.90	6.2 (0.4–66.9)		10.31 ± 9.38	7.1 (1–49.4)	
SCR (μmol/L)	153.86 ± 214.21	66 (28–1,655)		155.52 ± 244.11	65 (24–1949)	
eGFR (mL/min/1.73 m^2^)	104.16 ± 65.24	108.1 (2.6–332.9)		103.5 ± 62.32	106.1 (2.6–276.2)	
CLCr (mL/min)	88.91 ± 60.14	84.92 (3.5–295.5)		87.95 ± 58.66	82.72 (5.2–316.63)	
WBC (10^9/L)	8.80 ± 16.56	5.7 (0.1–154.2)		6.96 ± 6.03	5.35 (0.1–35.6)	
HB (g/L)	85.88 ± 20.71	84 (12–161)		84.33 ± 20.83	80 (28–162)	
PLT (10^9/L)	126.56 ± 124.88	85.5 (1–759)		144.02 ± 133.13	105 (7–605)	
Time after the last dose of biapenem administration (h)	6 ± 2.23	6 (0.5–20.73)		5.83 ± 2.84	5.95 (1–20.3)	
Immunocompromisation			34 (13.9)			11 (15.3)
Septic shock			66 (26.9)			20 (27.8)
Primary infection site						
Respiratory			108 (44.1)			29 (40.3)
Intra-abdominal			119 (48.6)			36 (50.9)
Other			18 (7.3)			7 (9.7)

ALT, alanine aminotransferase; AST, aspartate transaminase; AKP, alkaline phosphatase; GGT, glutamyltransferase; TB, total bilirubin; DB, direct bilirubin; TP, total protein; ALB, albumin; BUN, blood urea nitrogen; SCR, serum creatinine; eGFR, estimated glomerular filtration rate; CLCr, creatinine clearance; WBC, white blood cell count; HB, hemoglobin; PLT, platelet.

### 3.2 Model building

The minimum OFV indicated that the a two-compartment model (679.233) better described the data than a one-compartment model (734.914), so the pharmacokinetics of BPM were described by a two-compartment model with first-order elimination. Detailed covariate screening process is presented in Supplementary Table S1 in the Supplementary Material. The residual unexplained variability was best described by an additive residual error model. During the forward inclusion process of covariate screening, it was found that CLCr and ALB had a significant impact on total clearance (CL), while BUN was a significant covariate for intercompartment clearance (Q). In the backward elimination process, ALB was removed from the model. The OFV of the final model was 550.508, which was 132.991 lower than the basic model. The PPK parameters of the base and final models are summarized in Table 2. The final model for BPM is represented by the following equation:

$$CL\left(L/h\right)=8.33\times \left\{1+0.046\times \left[CLCr\left(ml/\min\right)-78.2\right]\right\}$$

$$V1\left(L\right)=13.4$$

$$Q\left(L/h\right)=3.75\times \left\{1+0.112\times \left[BUN\left(mmol/L\right)-6.8\right]\right\}$$

$$V2\left(L\right)=60.4$$

**TABLE 2:** Population pharmacokinetic parameter estimates of the final model and bootstrap.

Parameter	Final model		Bootstrap		
	Esteimate	RSE (%)	Median	5th-95th	Bias (%)
CL (L/h)					
CL = θ_1_×[1+θ_5_×(CLCr −78.2)]					
θ_1_	8.33	6.4	8.331	7.48–9.92	0.01
θ_5_	0.0046	11.9	0.0049	0.0034–0.0064	6.52
V1(L) = θ_2_					
θ_2_	13.4	15	13.23	10.94–23.55	−1.27
Q (L/h) = θ_3_×[1+θ_6_×(BUN -6.8)]					
θ_3_	3.75	13.6	3.58	2.26–5.73	−4.53
θ_6_	0.112	1.9	0.114	0.034–0.15	1.79
V2(L) = θ_4_					
θ_4_	60.4	17.5	64.72	23.38–169.07	7.15
Inter-individual variability					
ω_CL_	0.0591	19.8	0.057	0.031–0.088	−3.55
ω_Q_	1.12	25.2	1.10	0.26–2.94	−1.79
Residual variability					
ε	0.591	17.1	0.59	0.39–0.85	−0.17

RSE, relative standard error; CL, clearance (L/h); Q, inter-compartmental clearance (L/h), V1, volume of distribution of central compartment (L), V2, volume of distribution of the peripheral compartment (L); CLCr, creatinine clearance (mL/min), BUN, blood urea nitrogen (mmol/L); Bias(%)= (bootstrap estimated value-final model estimated value)/final model estimated value ×100%.

### 3.3 Model evaluation

#### 3.3.1 Internal evaluation

**FIGURE 1:**
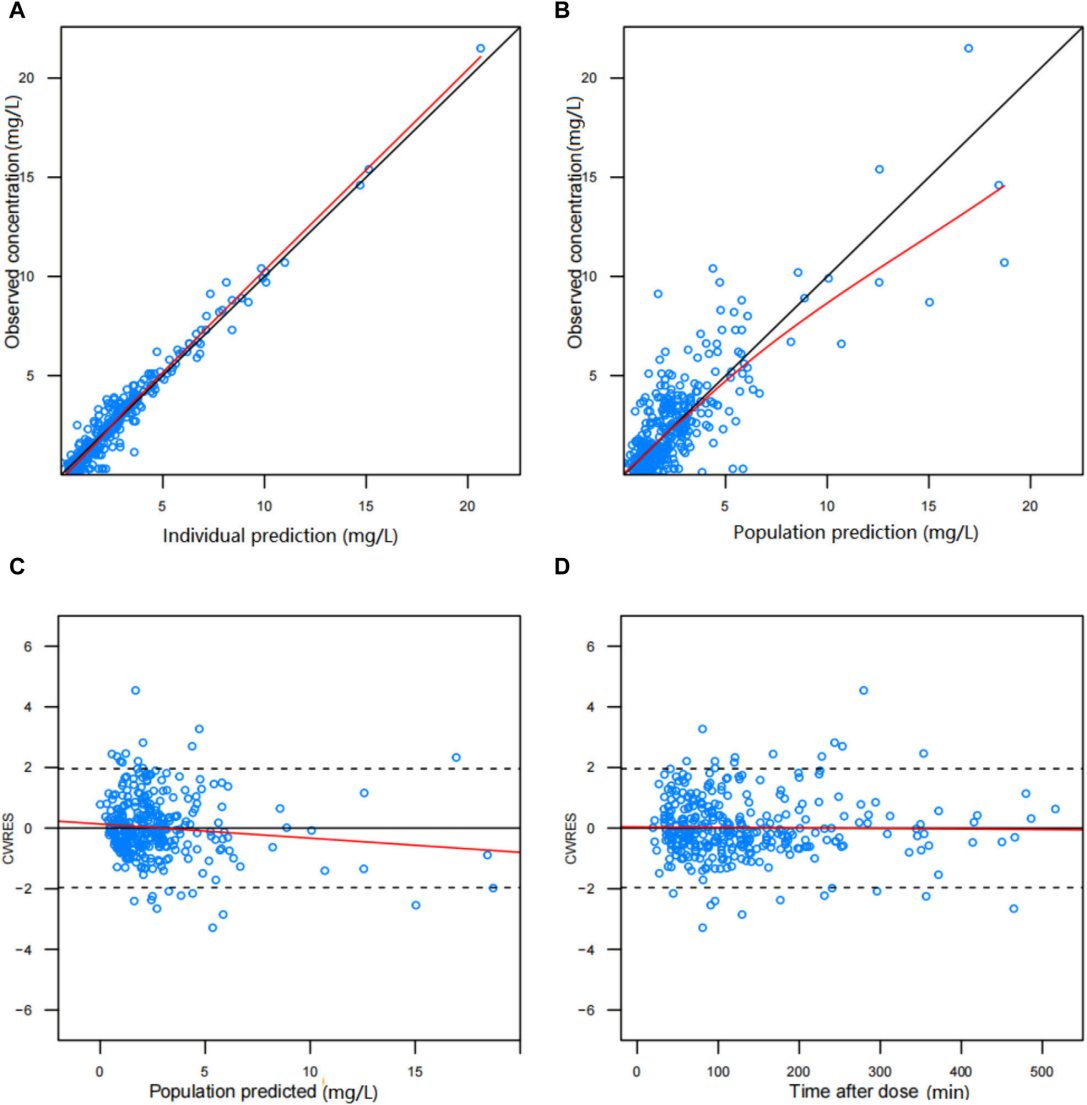
Diagnostic scatter plots of final population PK model for biapenem: **(A)** predicted vs. observed concentration, **(B)** individual predicted vs. observed concentration, **(C)** conditional weighted residual (CWRES) vs. predicted concentration, and **(D)** CWRES vs. time.

**FIGURE 2:**
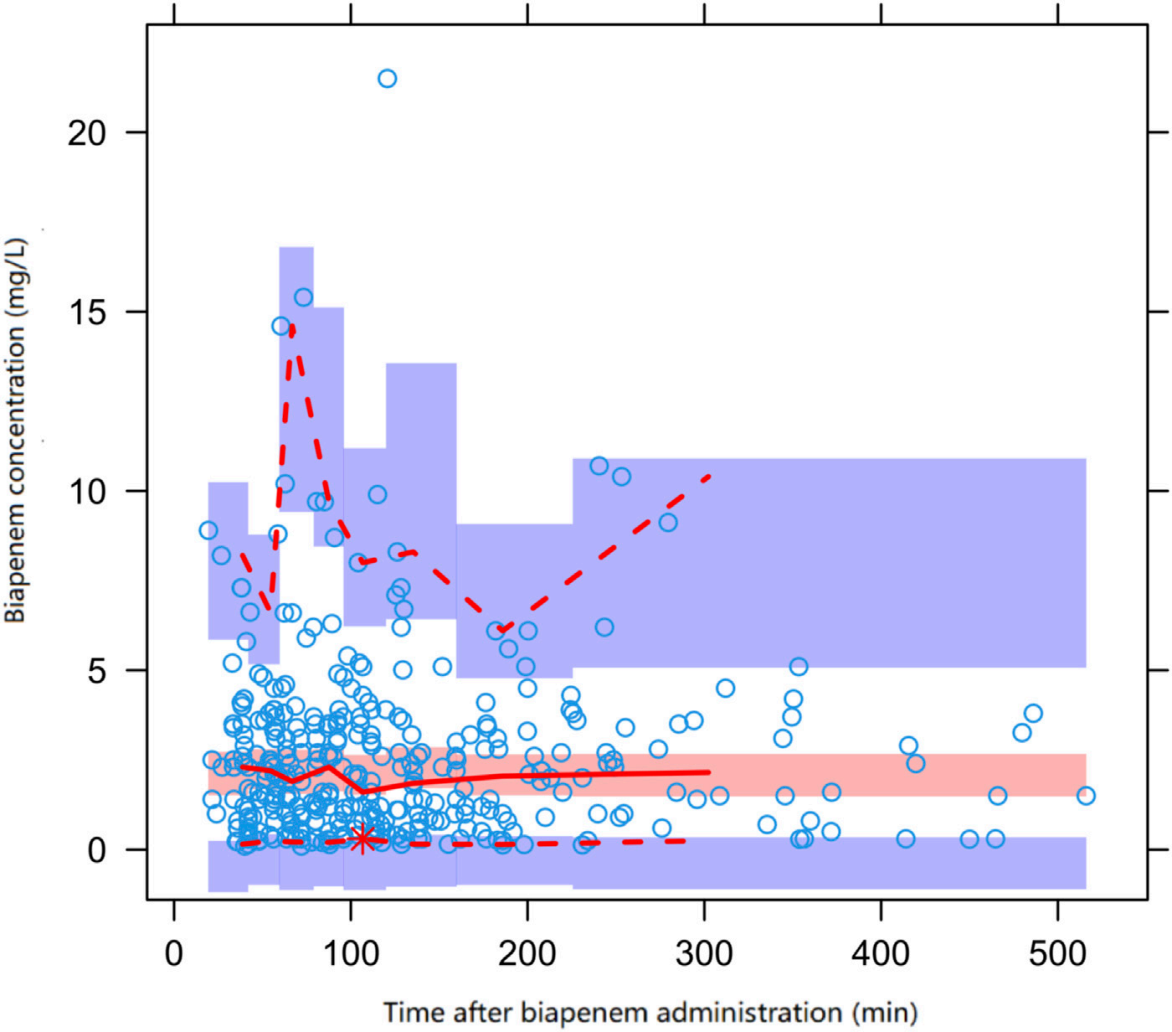
Prediction corrected visual predictive check (pcVPC) of biapenem concentration vs. time for the final model. Open circles represent observed concentrations, the solid line and the dashed line represent the median and the 95% CI of observations, respectively. The middle red shadow areas represent the 95% CI of the median for the results of 1,000 simulations of the final model and the blue shadow areas represent the 95% CI of the 10th and 90th percentiles of the results of 1,000 simulations of the pharmacokinetic final model.

The diagnostic plots demonstrated acceptable goodness of fit for the final PPK model of BPM. As depicted in Figure 1, there is no apparent systematic bias. Figure 1A shows the DV versus PRED scatter plot and Figure 1B shows the DV versus IPRED scatter plot. The diagnostics of WRES versus time as well as PRED are presented in Figures 1C, D, showing no obvious bias. The red solid line is the trend line of the scatter plot, and the black solid line is the reference line. As shown in Figures 1A, B, the trend lines of population predictions and individual predictions of the model are close to the reference line of Y = X, and the scattered points are evenly distributed on both sides of the reference line, indicating that the model individual predicted values are basically the same as the observed values. The scatter plot of weighted residuals is evenly distributed on both sides of 0, and most of the values are within ±2, indicating a good fit of the final model. The VPC results of the final model are shown in Figure 2. The curves from top to bottom represent the 95th, 50th, and 5th percentiles of observed concentrations, and the shaded area represents the 95% CI of simulated concentrations. The scatter points represent the observed values. According to the analysis, the proportion of observed concentrations falling within the predicted concentration 95% CI of the final model was 93.73%, and the percentile distribution of observed concentrations was similar to the 95% CI of the simulated data. Therefore, the final model has good predictive performance.

#### 3.3.2 External evaluation

A total of 115 BPM concentration samples from 72 adult patients were included in the external evaluation cohort of the final model. The results of the prediction error test method were as follows: MDPE = 6.75%, MAPE = 26.25%, F_20_ = 42.61%, and F_30_ = 53.04%. The prediction error results were within the target range of standards, indicating that the final model has good predictive performance.

### 3.4 Microbiology and clinical outcomes

Of the 317 patients, at least on major pathogen was identified in 211 patients (67%) and included in the microbiology and clinical outcome analysis. A total of 265 pathogens were identified and the microbiological findings are presented in Table 3. The most commonly isolated microorganisms were *Escherichia coli* (25%), *Klebsiella pneumoniae* (15%), *Pseudomonas aeruginosa* (15%), and *Acinetobacter baumannii* (12%). The median MIC values of BPM for *Enterobacteriaceae*, *P. aeruginosa*, and *A. baumannii* were 0.25, 1.0, and 8.0 mg/L, respectively.

**TABLE 3:** Microbiologic characteristics (265 isolates from 211 patients) and biapenem susceptibility.

Pathogen	No. of isolates	Biapenem MIC range (mg/L)
*Escherichia coli*	66	0.1–2
*Klebsiella pneumoniae*	31	0.5–2
*Klebsiella pneumoniae* (CRE)	9	≥16
*Pseudomonas aeruginosa*	37	1–8
*Pseudomonas aeruginosa* (CR-GNB)	3	≥16
*Acinetobacter baumannii*	9	1–2
*Acinetobacter baumannii* (CR-GNB)	23	≥16
*Enterobacter cloacae*	13	0.25–0.5
*Enterococcus faecalis*	29	1–2
*Enterococcus faecium*	10	ND
*Stenotrophomonas maltophilia*	5	ND
*Staphylococcus aureus*	5	ND
*Staphylococcus* spp.	4	ND
*Serratia marcescens*	5	0.25
Other *Enterobacteriaceae*	11	0.1–2
Other *Acinetobacter* spp.	2	1–8
Other *Pseudomonas* spp.	3	1–4

CRE, carbapenem-resistant *Enterobacteriaceae*; CR-GNB, carbapenem-resistant Gram-negative bacteria; ND, not determined.

Overall, 125 (59%) patients achieved successful clinical cure after BPM treatment. The baseline clinical characteristics are shown in Table 4. Among all the patients, 85% (179/211) of them achieved 40% *fT*_>MIC_ and 60% (127/211) of them achieved 70% *fT*_>MIC_. The proportion of patients reaching the *fT*_>MIC_ targets were higher in the clinical success group than in the failure group. The results of the multivariable logistic analyses of clinical success are presented in Table 5. According to the analyses, 70% *fT*_>MIC_ was associated with an increasing possibility to achieve clinical cure when treating septic patients with BPM. The Odds Ratio (OR) was resulted to be 7.07 with a 95% confidence interval of 2.45–20.37 and a *p*-value <0.001. Also, hypoalbuminemia is a risk factor for treatment failure (OR: 0.33, 95% CI: 0.12–0.91, *p*-value: 0.03).

**TABLE 4:** Comparison of baseline characteristics between clinical success and failure groups.

Characteristics	Success (n = 125)	Failure (n = 86)	*p*-value
Male (%)	82 (66)	59 (69)	0.65
Age (years)	62.12 ± 16.62	60.05 ± 15.88	0.52
Body weight (kg)	62.91 ± 13.61	63.93 ± 7.63	0.61
Albumin (g/L)	30.00 ± 3.54	28.13 ± 3.39	0.02
Septic shock (%)	30 (24)	28 (33)	0.17
Serum creatinine (μmol/L)	93.50 (61.00, 450.25)	120.50 (67.75, 409.75)	0.67
White blood cell count (10^9/L)	10.40 (7.13, 14.58)	12.30 (8.85, 18.58)	0.15
C-reactive protein (mg/L)	88.09 (35.50, 129.85)	71.55 (44.75, 116.78)	0.93
Target attainment (%)			
40% *fT* > MIC	108 (86)	71 (83)	0.45
70% *fT* > MIC	96 (77)	31 (36)	<0.001
Therapy regimens (%)			
Concomitant antimicrobials	59 (47)	44 (51)	0.57
Biapenem monotherapy	66 (53)	42 (49)	
Primary infection site (%)			
Respiratory	55 (44)	38 (44)	0.81
Intraabdominal	56 (45)	36 (42)	
Other	14 (11)	12 (14)	

**TABLE 5:** Multivariable Logistic analysis of factors associated with biapenem treatment clinical success.

Variables	Odds ratio	95% Confidence interval	*p*-value
70% *fT* > MIC	7.07	2.45–20.37	<0.001
Hypoalbuminemia	0.33	0.12–0.91	0.03

### 3.5 Dosing regimen optimization

**FIGURE 3:**
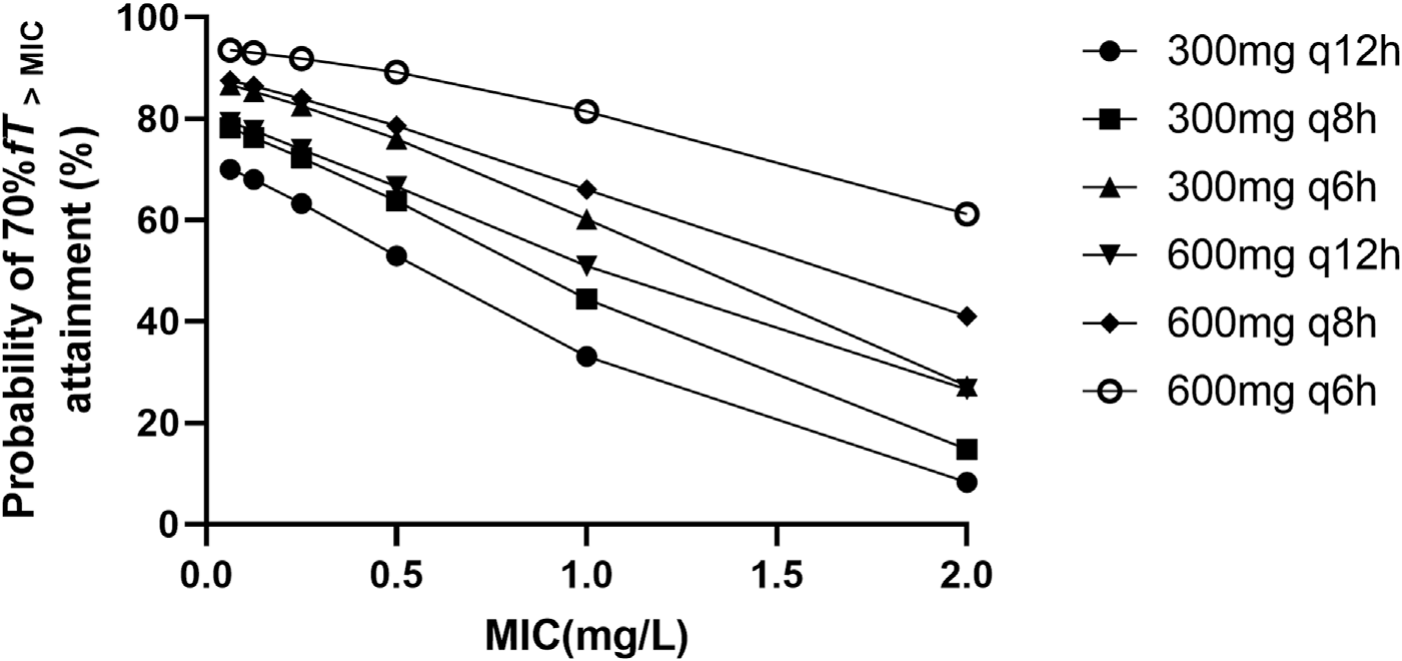
Probability of ≥70% fT > MIC attainment for different biapenem dosage regimens with different MIC values ranging from 0.0625 to 2 mg/L.

The target attainment (70% *fT*_>MIC_) rate as functions of the simulated dose for different MIC susceptibility breakpoints are shown in Figure 3. The results of the simulation revealed that the existing standard dosage regimens, as delineated in the product label (up to a maximum of 1.2 g per day), are insufficient to achieve a PTA of 70% *fT*_>MIC_ in more than 60% septic patients when MIC is higher than 1 mg/L. Within the dosage regimen recommended by the current product label, the administration regimen of 300 mg every 6 hours appears to be the optimal choice for patients with sepsis. However, this regimen may not provide adequate antimicrobial activity when there is an increase in the MIC. When BPM is administered 300 mg every 6 h, only 60.22% of the septic patients achieved the pharmacodynamic target for MIC of 1 mg/L, showing that this was an underdose. When a maximum dose of 2.4 g per day was administered (600 mg every 6 h), 81.47% of the septic patients achieved the pharmacodynamic target for MIC of 1 mg/L. In summary, the BPM regimens needed to achieve optimal PTA for 70% *fT*_>MIC_ is 600 mg every 6 h, the recommended daily dose of product label may not be sufficient in septic patients.

## 4 Discussion

Life-threatening sepsis and severe nosocomial bacterial infections in hospitalized patients represent a significant clinical challenge (Evans et al., 2021). A robust correlation exists between the *fT*_>MIC_ of carbapenems and their clinical efficacy (Zhanel et al., 2007). Deviations from optimal dosage, whether insufficient or excessive, can lead to adverse effects, consequently influencing the overall clinical efficacy of these antimicrobial agents (Boonpeng et al., 2022; Dinh et al., 2022). Consequently, this research endeavors to develop a BPM PPK model specifically for adult patients suffering from sepsis. This initiative is intended to furnish empirical support for the optimization of clinical administration strategies of this antimicrobial agent.

At present, there are many methods evaluating the influence of covariates on PK model parameters. In spite of the selected method, the intention of such evaluations is usually to describe inter- and intra-subject variability in PK parameters with patient factors. These factors may be those patient characteristics that are recorded for each subject at a baseline study visit and describe patient-specific factors such as the subject’s gender, age, weight, and height, for instance. Covariates that describe something about the current disease status and/or medical history for each patient may also be collected (Mould and Upton, 2013). In longer term studies, the extent to which a covariate, such as renal function, measured by creatinine clearance, may be changing could be particularly important to capture, especially BPM elimination is largely renal (Perry and Ibbotson, 2002). Other covariates that are sometimes studied as time-varying factors include laboratory measures of hepatic function, which may be measured at multiple study visits, as well as concomitant medications of interest with the potential to induce a drug–drug interaction. So the above covariates were introduced in the model building in this study.

The present study used a two-compartment model to describe the pharmacokinetic data of BPM, which is consistent with previous study (Ikawa et al., 2008b). To assess the pharmacokinetic characteristics of the adult patients with sepsis, we examined the impact of covariates on pharmacokinetic parameters. The previous study of Ikawa et al. found that the average CL of BPM in adult patients was 8.13 L/h for CLCr of 77.5 mL/min (Ikawa et al., 2008b). In this study, an average CL was observed, the mean CL of BPM in patients with sepsis was 8.33 L/h for CLCr of 78.2 mL/min. Hang et al. reported an average CL BPM in critically ill patients was 20.9 L/h, higher than the CL observed from this study (Hang et al., 2018). Although ALB was included as a covariate affecting CL during the model building process using forward inclusion method, it was eventually eliminated from the final model. Therefore, it was found through the study that CLCr was the most significant influencing factor on BPM CL. However, we speculate that a low ALB level may also have a potential impact on CL of BPM. Patients with lower level of ALB was often related with impaired renal or liver function. A previous study has also shown that, under the same renal function level, patients with decompensated cirrhosis have relatively lower meropenem trough concentrations, suggesting that alteration of pharmacokinetics was common in patients with impaired liver function, which could be similar in pharmacokinetics of BPM (Bastida et al., 2020).

Also, the volume of distribution of BPM differs significantly from previous studies. Ikawa et al. reported an average V (V_1_+V_2_) of 14.3 L in adult patients (Ikawa et al., 2008b) while Hang et al. found the V was 46.43 L in critically ill patients (Hang et al., 2018). In the current investigation, the V (V_1_+V_2_) of BPM was observed to reach a notable 73.8 L among patients suffering from sepsis, approximately four to five times higher than that observed in other patient populations in previous studies. Although only a few studies have investigated the population pharmacokinetics of carbapenems in septic patients, an increase in the volume of distribution is common. Fukumoto et al. revealed the volume of distribution of meropenem V (V_1_+V_2_) in patients with sepsis was 39.7L (Fukumoto et al., 2023), while Murínová et al. reported an average V of 55L in patients with serious infection (Murínová et al., 2022), respectively. Both studies reported higher V of meropenem in patients with sepsis comparing with other patients, which may be caused by the increase in V of hydrophilic drugs, which is quite common in critically ill patients (Roberts and Lipman, 2006). In this study, the observed elevation in the V of BPM among septic patients, also a hydrophilic drug, is consistent with the theory established in previous research.

In the present study, we found that the Q of BPM in septic patients is affected by the levels of BUN. It has been reported that BUN levels are significantly correlated with the prognosis of sepsis patients (Li et al., 2021; Harazim et al., 2023), with even a mild elevation in BUN being associated with an increased mortality in these patients. While the association between elevated BUN levels and neurohormonal response has predominantly been established within populations of patients experiencing cardio-renal issues, we contend that elevated BUN, irrespective of eGFR, may intricately signify intricate underlying pathological processes directly involved in the pathophysiology of sepsis. Although the link between elevated BUN and neurohormonal response has almost exclusively been derived from population of patients with cardio-renal problems we believe that elevated BUN independent of GFR may sensitively reflect complex underlying pathological processes directly implicated in the pathophysiology of sepsis. In the context of sepsis, arterial underfilling arising from systemic inflammation-induced arterial vasodilation serves as a robust trigger for the activation of the sympathetic nervous system (SNS), the renin–angiotensin–aldosterone axis (RAAS), and the non-osmotic release of vasopressin (AVP) (Gomes et al., 2020). While the SNS, RAAS and AVP all constitute pivotal adaptive responses to stress, there is a compelling inclination to hypothesize that excessive and prolonged activation during sepsis may transition into a maladaptive state, eliciting adverse effects. Moreover, sepsis is distinguished by a profound and frequently enduring catabolic state, culminating in the depletion of muscle mass and neuromuscular weakness. In this context, elevated BUN may additionally function as an indicator of severe catabolism in sepsis. Substantiating this concept, Haines et al. recently recognized the urea-to-creatinine ratio as a promising biomarker of catabolism associated with critical illness (Haines et al., 2019). The aforementioned alterations may signify the undiscovered prognostic value of BUN in the pathophysiology of sepsis, potentially entwined with systemic metabolism and inflammatory levels, which influence the pharmacokinetics of BPM.

According to our analyses, achieving a 70% *fT*_>MIC_ significantly enhances the likelihood of clinical cure in patients with sepsis treated with BPM. This finding underscores the critical role of optimizing antimicrobial exposure to improve therapeutic outcomes. Additionally, the analysis identified hypoalbuminemia as a significant risk factor for treatment failure. The implications of these findings are twofold. Firstly, they highlight the importance of individualized dosing strategies based on PK/PD principles to maximize the efficacy of BPM in treating septic patients. Secondly, the identification of hypoalbuminemia as a risk factor suggests that addressing underlying nutritional deficits or managing the protein distribution profiles may be crucial in enhancing treatment efficacy. These insights pave the way for targeted interventions and optimized therapeutic strategies in the management of sepsis, potentially improving patient outcomes through tailored therapeutic approaches.

Carbapenems demonstrate time-dependent antibacterial activity, wherein their efficacy is most closely associated with the % *fT*_>MIC_. It is generally recommended that the %*fT*_>MIC_ of carbapenems should reach a threshold of 40%–50% to achieve optimal bactericidal effects, and the position paper of European Society of Intensive Care Medicine recommended a clinical PK/PD target for efficacy of 50%–100%*fT*_>MIC_ (Abdul-Aziz et al., 2020). In this study, we chose a target of 70% *fT*_>MIC_. Although there are limited pharmacodynamic data of BPM beyond this study, a goal of 70% *fT*_>MIC_ would represent a more conservative endpoint. As shown in the results of Monte Carlo simulations, in the population of patients with sepsis, it appears that the recommended dosage as per the product label may not adequately achieve the clinical efficacy targets. Under the daily total dose regimen of 1.2 g, probability was lower than 80% to attain 70% *fT*_>MIC_ in patients with pathogens exhibiting a MIC of 1.0 mg/L. Only the 300 mg q6h regimen demonstrates a probability exceeding 60% for attaining 70% *fT*_>MIC_ in this microbial subset. The probability drop to less than 30% in patients with pathogens exhibiting a MIC of 2.0 mg/L under the daily total dose regimen of 1.2 g. This implies that a higher daily dosage of BPM is required in septic patients to achieve therapeutic goals. To improve PK/PD target attainment, an optimized dosing regimen of 600 mg q6h was required for pathogens with a MIC of 1.0 mg/L because of a higher PD parameter attainment (81.47%). In septic patients, the BPM regimen of 600 mg q8h appears to exhibit no significant increase in the probability of achieving PK/PD targets compared to the 300 mg q6h regimen. The difference in target attainment probabilities between the two dosage regimens exceeds 10% only when the MIC is 2 mg/L (41.08% vs. 27.32%). Currently, there are no recommendations published by CLSI and EUCAST regarding bacterial susceptibility breakpoints for BPM. According to the antimicrobial susceptibility report from the bacteriological laboratory in China, *Escherichia coli* and *Enterobacter cloacae* exhibited susceptibility to BPM, with MIC_90_ values of 0.25 mg/L and 0.5 mg/L, respectively, while *Klebsiella pneumoniae* has an MIC_90_ of 2 mg/L (Dong et al., 2016). Therefore, based on our research findings, it is suggested that BPM may necessitate a daily dosage higher than the current clinical standard (1.2 g per day) in septic patients to achieve sufficient clinical efficacy while infections are caused by *Klebsiella pneumoniae*. This calls for further exploration through meticulously designed prospective randomized controlled clinical studies.

At the same time, we must confess that the current study’s limitation to a single-center cohort restricts its external validity. Extending the future study to include multiple centers, especially those in different geographic locations, would not only enhance the robustness of the pharmacokinetic model but also improve its generalizability across broader patient populations. While the study effectively identifies creatinine clearance and blood urea nitrogen as significant predictors of biapenem clearance, these findings scratch the surface of the complex interplay of factors impacting drug metabolism in septic patients. Investigating additional covariates, such as genetic factors, could uncover nuances in pharmacokinetic parameters that standard analyses might overlook. The inclusion of a validation cohort in the study is commendable; however, validation through an independent cohort from diverse demographics or geographic areas would solidify the pharmacokinetic model’s utility and reliability. This approach would mitigate the cohort-specific bias and confirm the model’s efficacy across various clinical settings. The manuscript delineates the inter-individual variability observed in the pharmacokinetic parameters of biapenem. Identifying the sources of this variability is crucial for optimizing dosing strategies. Moreover, exploring inter-occasion variability, which accounts for changes in pharmacokinetic parameters between different visits of the same patient, could enhance the model’s accuracy. This variability might be attributed to changes in disease state, concomitant medications, or recovery phase, and its inclusion in the analysis could refine dose adjustments over the course of treatment. However, due to the retrospective nature of the study, we were not able to discover the variability more specifically. This study fail to integrate microbiological efficacy data, such as bacterial load dynamics and susceptibility changes, to enhance the pharmacokinetic model’s utility by linking drug exposure to microbial response. This approach should be utilized in future prospective study to provide a more holistic understanding of biapenem’s therapeutic impact, particularly in modifying treatment strategies based on microbiological feedback. Lastly, while the manuscript adeptly discusses pharmacokinetic-driven dose optimization using Monte Carlo simulations, and compare the clinical outcomes of the, integrating these theoretical dosing schemes with real-world clinical outcomes would vastly improve the practical value of the study. Prospective clinical trials designed to evaluate the correlation between optimized dosing regimens and patient-centric outcomes such as infection resolution and survival rates are essential for translating pharmacokinetic insights into clinical practice.

This study represents the first exploration of the PK parameters of BPM in septic patients. The considerable sample size included in this study can be attributed to the routine implementation of TDM in clinical practice for BPM therapy. There are limitations in our present study. The use of data collected during routine TDM limiting the number of BPM concentrations measured for each patient. Due to the substantial heterogeneity in microbiota among septic patients and the complexity of clinical conditions during treatment, integrating PPK models, actual clinical efficacy, and PK/PD index is challenging and complicated. Ultimately, the optimal dose regimen based on modeling and simulation should be evaluated in clinical practice to confirm its clinical benefits.

## 5 Conclusion

The PPK model of BPM was developed in patients with sepsis and elucidated the significant effects of CLCr and BUN on BPM pharmacokinetics. The target of 70%*fT*_>MIC_ is associated with favorable clinical outcomes_._ The current daily dosage regimen of 1.2 g may potentially fall short of achieving sufficient clinical efficacy in septic patients when treating pathogens with MIC>1 g/L. To attaian 70%*fT*_>MIC_, the dosage regimen of 600 mg every 6 h appears to be the optimal choice. These results better define the pharmacokinetics of BPM and help in the choice of the appropriate dosage regimens of BPM for patients with sepsis.

## Data Availability

The raw data supporting the conclusion of this article will be made available by the authors, without undue reservation.
