# Supplementary material for: Population pharmacokinetics, dosing optimization and clinical outcomes of biapenem in patients with sepsis
Source: Front Pharmacol. 2024 May 10;15:1388150. doi: 10.3389/fphar.2024.1388150 (PMC11116716; doi:10.3389/fphar.2024.1388150)
Supplement: Supplementary file 1 [file Table1.docx]

Table S1 Population pharmacokinetic parameter screening process of the final model

| Variable | Inclusion | | Elimination | |
| --- | --- | --- | --- | --- |
|  | OFV | △OFV | OFV | △OFV |
| Base model | 683.499 |  |  |  |
| CLCr on CL | 550.508 | -132.991 | 605.716 | 75.634 |
| BUN on Q | 530.082 | -20.425 | 550.508 | 20.425 |
| ALB on CL | 526.191 | -3.891 | 526.159 | 0.285 |
| Final model |  |  | 550.508 |  |

OFV, objective function value; CL, clearance (L/h); Q, inter-compartmental clearance (L/h), CLCr, creatinine clearance (ml/min), BUN, blood urea nitrogen (mmol/L), ALB, albumin (g/L).
