# Supplementary figures and images for: Population pharmacokinetics, dosing optimization and clinical outcomes of biapenem in patients with sepsis
Source: Front Pharmacol. 2024 May 10;15:1388150. doi: 10.3389/fphar.2024.1388150 (PMC11116716; doi:10.3389/fphar.2024.1388150)

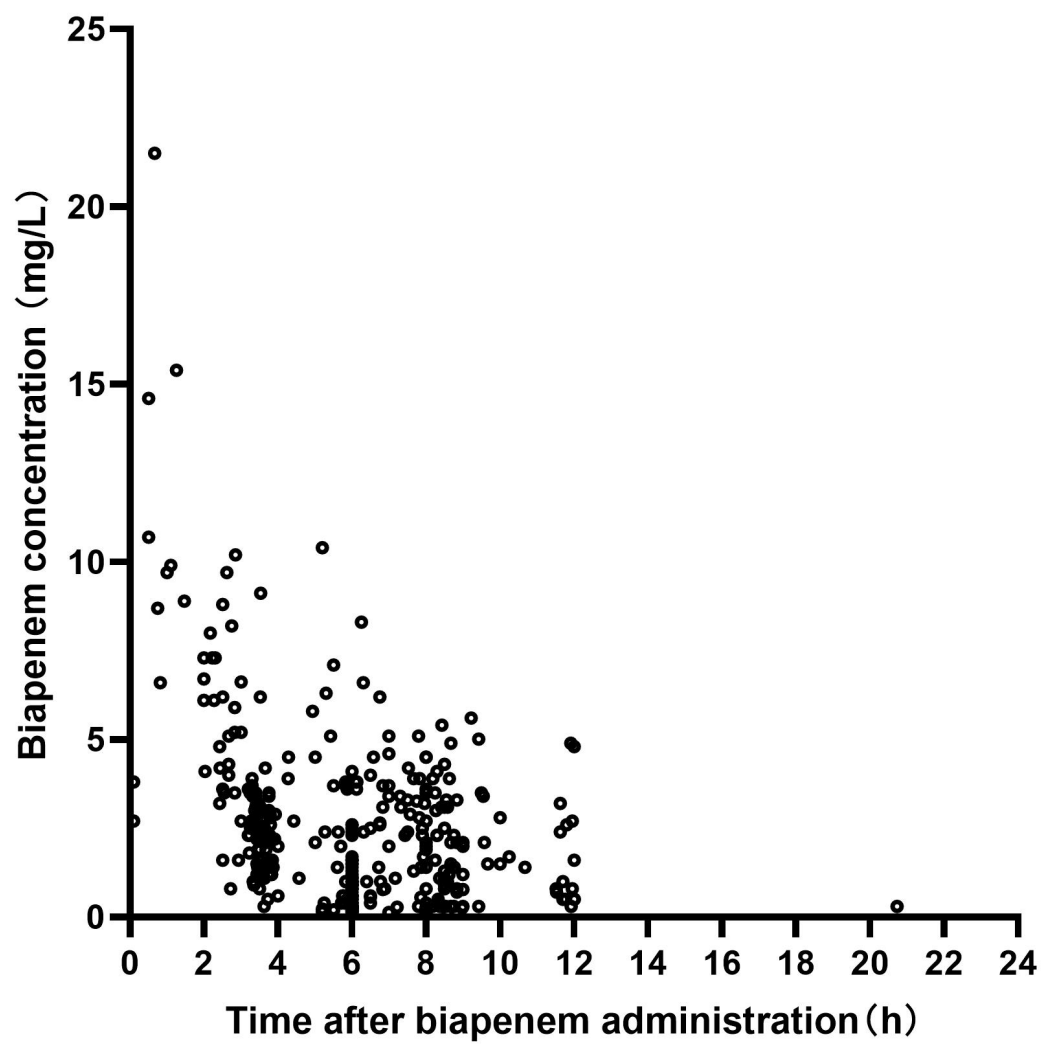

1

2 Figure S1 Scatter plots of biapenem concentration vs. time (n = 351).

Supplement: Supplementary file 2 [file Image1.PDF]
